# Passing Cerclage Wires Through a Direct Anterior Approach in Total Hip Arthroplasty: Surgical Technique

**DOI:** 10.1016/j.artd.2026.102146

**Published:** 2026-09-18

**Authors:** Nikhil Vallabhaneni, Utkarsh Anil, Allan Metz, Jeremy M. Gililland, Lucas A. Anderson

**Affiliations:** Department of Orthopaedic Surgery, University of Utah, Salt Lake City, UT, USA

**Keywords:** Intraoperative fracture management, Anterior approach, Cerclage fixation, Iatrogenic vascular injury, Soft-tissue protection

## Abstract

Passage of cerclage wires, either for prophylactic stability or for intraoperative fracture fixation, is a commonly used technique in total hip arthroplasty. However, the same techniques for wire placement cannot be used regardless of approach. In the direct anterior approach, common methods to pass wires may place the lateral femoral circumflex artery perforators at risk, as well as the proximal and distal neurovascular bundles to the vastus lateralis. We describe a technique for passing cerclage wires with the direct anterior approach that minimizes neurovascular injury.

## Introduction

**Figure 1:**
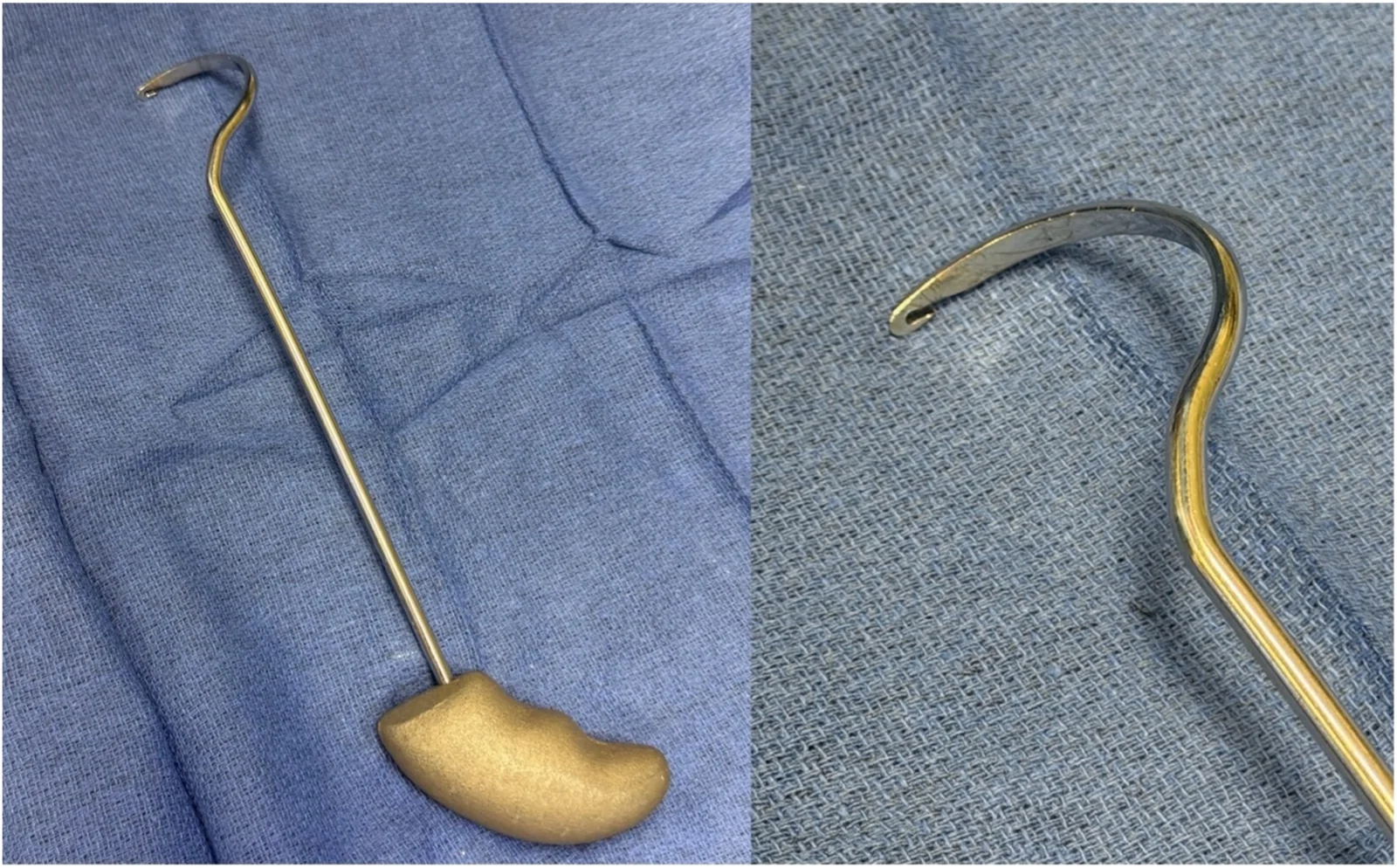
Traditional Luque wire hook passer demonstrating the hooked tip design prone to snagging LFCA perforating branches during cerclage passage.

Cerclage wiring is a valuable adjunct in total hip arthroplasty (THA) for prophylactic stabilization or to stabilize proximal femoral fractures and osteotomies. In the direct anterior (DA) approach to THA, however, passing a cerclage wire around the femur can be challenging because of limited exposure of the femur and the risk of injury to the proximal and distal neurovascular branches to the vastus lateralis, as well as to perforators of the lateral femoral circumflex artery (LFCA)—a significant source of bleeding and postoperative hematoma [1,2]. As a result, the conventional Luque wire passer, which features a hooked tip, is notoriously prone to snagging perforating branches of the LFCA (Fig. 1). There is a need for safer instruments and techniques to facilitate cerclage wiring in DA THA.

**Figure 2:**
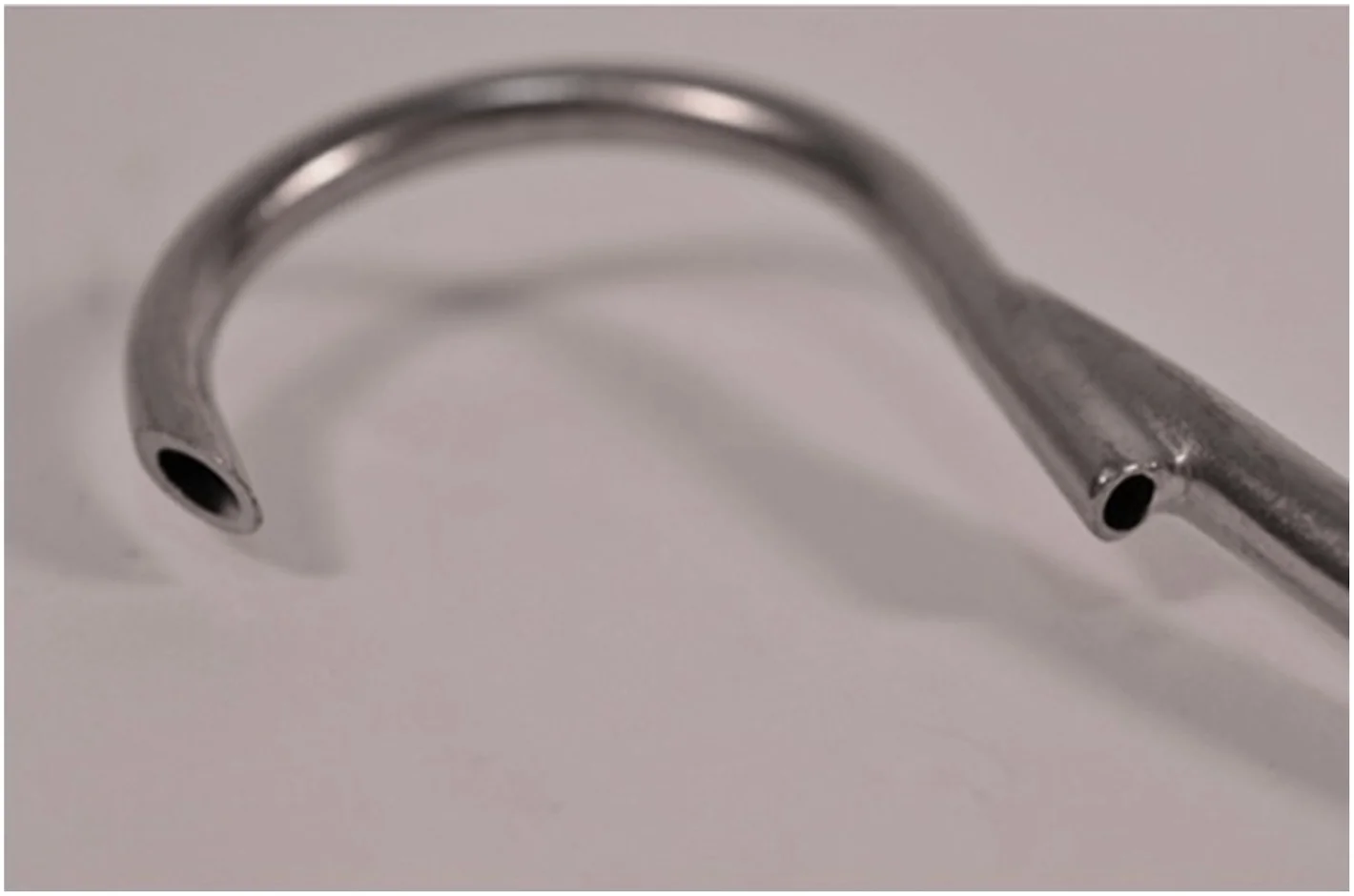
Blunted, hookless cable passer with a hole in its tip for wire passage, designed to minimize neurovascular injury.

Therefore, a blunt, nonhooked cable passer with a hole in its tip for the wire allows controlled passage without catching on soft tissues. An ideal cable passer should be sharp enough to penetrate intermuscular planes yet blunt enough to avoid tearing vessels, with a sturdy handle for twisting the wire and a sturdy neck that does not bend (Fig. 2). In addition to improved tools, alternative cerclage materials have been explored. Polyethylene cables (nonmetallic cerclage loops) have slight elasticity and can be tightened, loosened, and retightened, which may help in incremental tensioning. Kuster et al. [3] reported that an elastic polyethylene cable halted ∼30% of crack propagation in a cadaveric model, whereas a traditional metal cable halted ∼73%. While metal cables provide strong fixation, they can fret or corrode against implants and generate metal debris. Polymeric cables eliminate metal-on-metal contact at the cerclage interface, at the cost of lower stiffness. Standard stainless-steel wires are monofilament implants that are secured by twisting rather than crimping. Clinical studies have shown that wires perform equivalently to cables in supporting fractures, with no differences in healing or implant stability. Regardless of the cerclage implant selected, the technique for passage around the proximal femur is the same, and a safe, reproducible technique for passage through the DA approach is the focus of this report.

In this guide, we describe the technique for cerclage wire passage through the DA approach using a blunt-tipped cable passer that passes the wire in a controlled fashion to avoid neurovascular injury. The procedure is straightforward and reproducible, allowing the surgeon to address femoral fractures or prophylactically stabilize the femur during DA THA without needing extensile exposure or additional incisions.

## Surgical technique

### Proximal cerclage technique

Place the patient supine on the operating table as per the standard DA THA. Position the operative leg in neutral rotation and extension initially. A neutral leg position relaxes the iliotibial band and anterior soft tissues, allowing for easier passage of instruments. Manipulation of the operative leg position during wire passage is essential, whether on a specialized table with one leg draped or on a regular operative table with both legs draped free.

**Figure 3:**
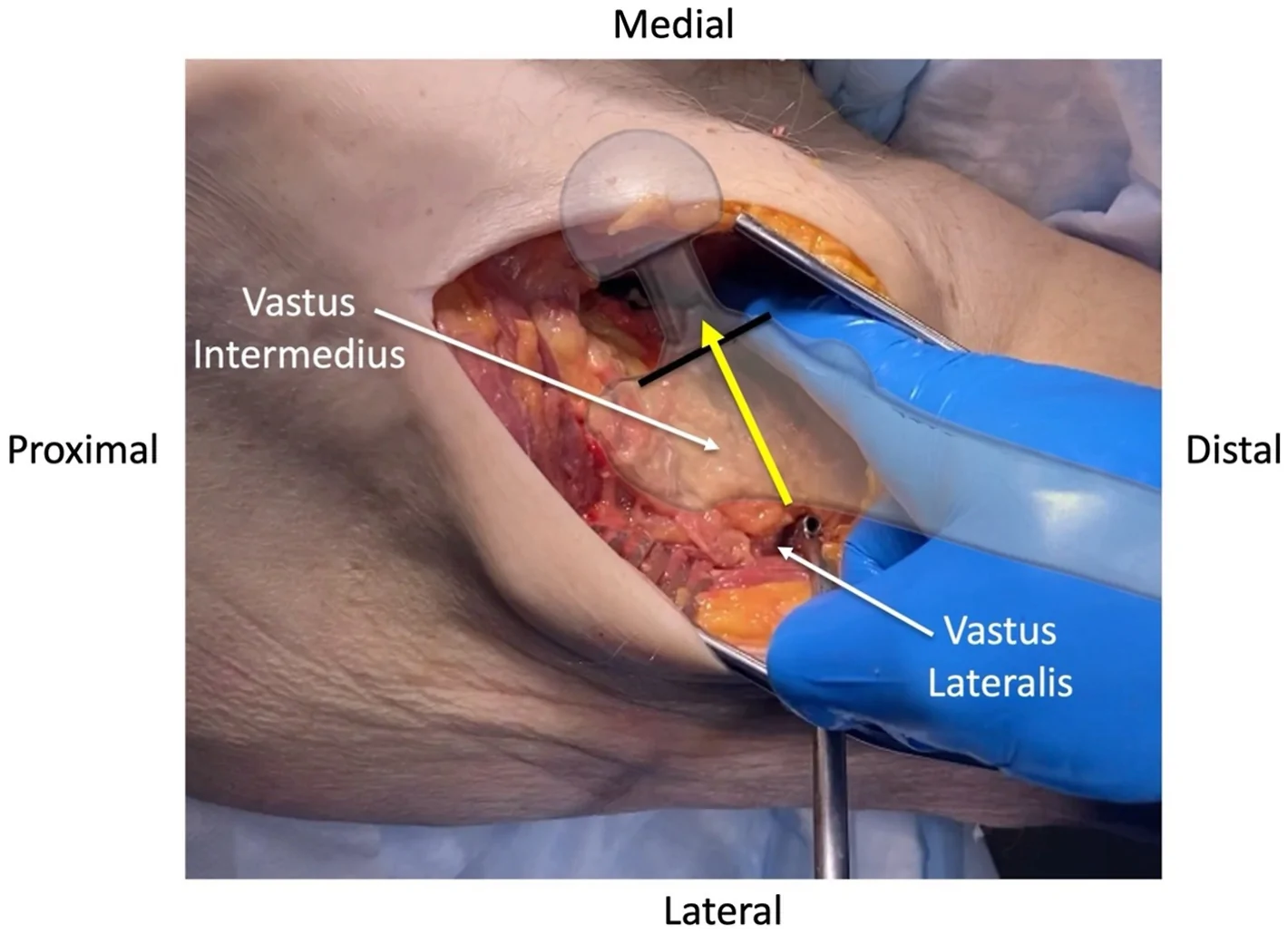
Intraoperative photograph demonstrating initial insertion of the cable passer from the lateral side of the proximal femur, advanced deep into the vastus lateralis along the lateral cortex.

**Figure 4:**
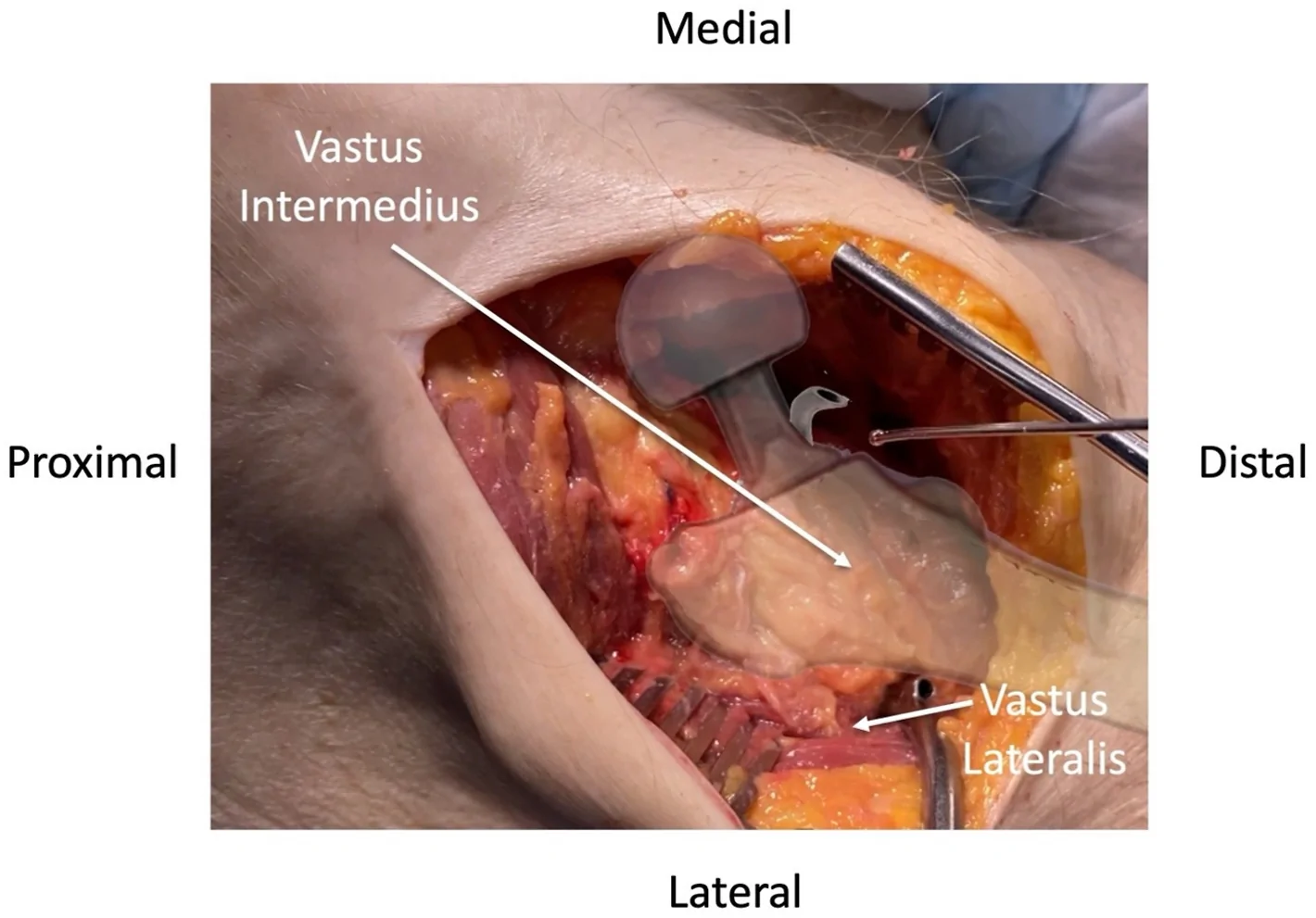
Intraoperative photograph demonstrating the cable passer tip exiting the medial aspect of the femur at the posterior calcar after passage around the posterior femur with the leg in external rotation.

**Figure 5:**
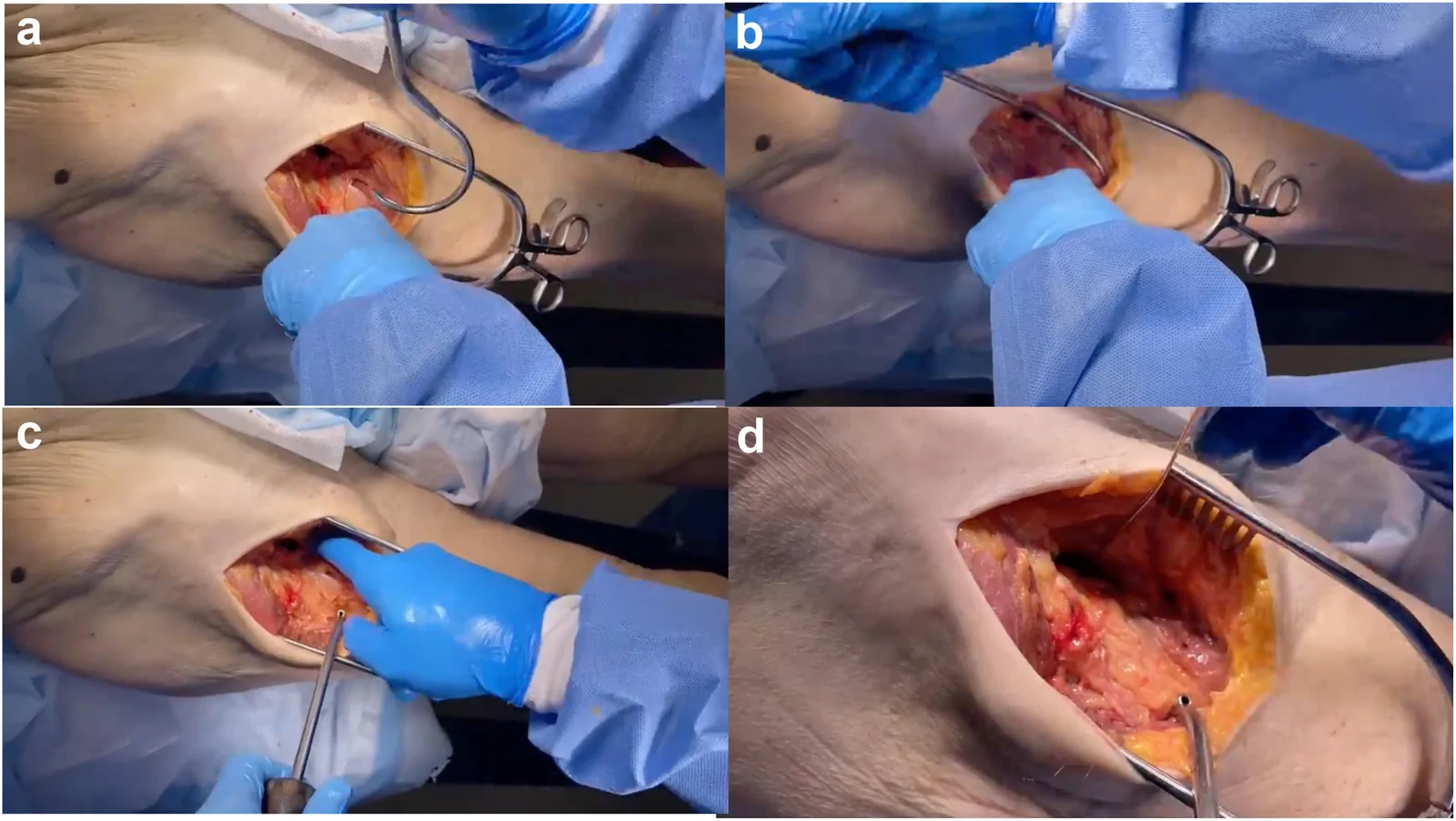
Proximal cerclage wire passage. (a) With the femur internally rotated to facilitate lateral exposure, the cable passer is introduced along the lateral aspect of the femur, deep to the tensor fascia lata. (b) The cable passer is advanced from lateral to medial around the posterior aspect of the femur, maintaining contact between its tip and the femoral cortex to avoid snagging soft tissues. (c) The femur is externally rotated, bringing the tip of the cable passer into view as it emerges through the posterior capsule on the medial aspect of the femur. (d) The selected cerclage wire or cable is passed through the cable passer from medial to lateral behind the femur.

Exposure to the lateral aspect of the proximal femur is created through the DA incision (Hueter interval). Identify the plane between the vastus lateralis and vastus intermedius muscle bellies, just below the vastus ridge. Use a curved tonsil clamp to develop this plane gently. The dissection should stay immediately adjacent to the femur, deep into the vastus lateralis. This is essentially a subvastus lateralis dissection from anterior trajectory, creating a path that the cable passer and wire can travel. Take care to stay on bone and avoid venturing too anterolateral to protect the descending branch of the LFCA or too posterior to avoid the profunda femoris perforators. Staying on the femoral cortex while dissecting will help safeguard nearby neurovascular structures. Adequate exposure of the lateral femur in this submuscular plane is critical for the subsequent passage of the wire. Insert the cable passer from the lateral side of the femur through the above dissection and advance it deep into the tensor fascia lata and vastus lateralis, hugging the lateral cortex of the femur (Fig. 3). Once the cable passer is around the posterior femur, externally rotate the operative leg to 90° of external rotation, thereby bringing the posteromedial calcar into view and aligning the trajectory for the passer. Aim the passer tip toward the midpoint of the posterior femoral neck cut, gently advancing the passer until its tip is visible at the posterior aspect of the calcar. The passer should penetrate the external rotators and posterior capsule at the level of the posterior calcar and should be in contact with the bone the entire way as this ensures it stays in the extra-periosteal plane to avoid neuromuscular or vascular damage (Figs. 4 and 5).

**Figure 6:**
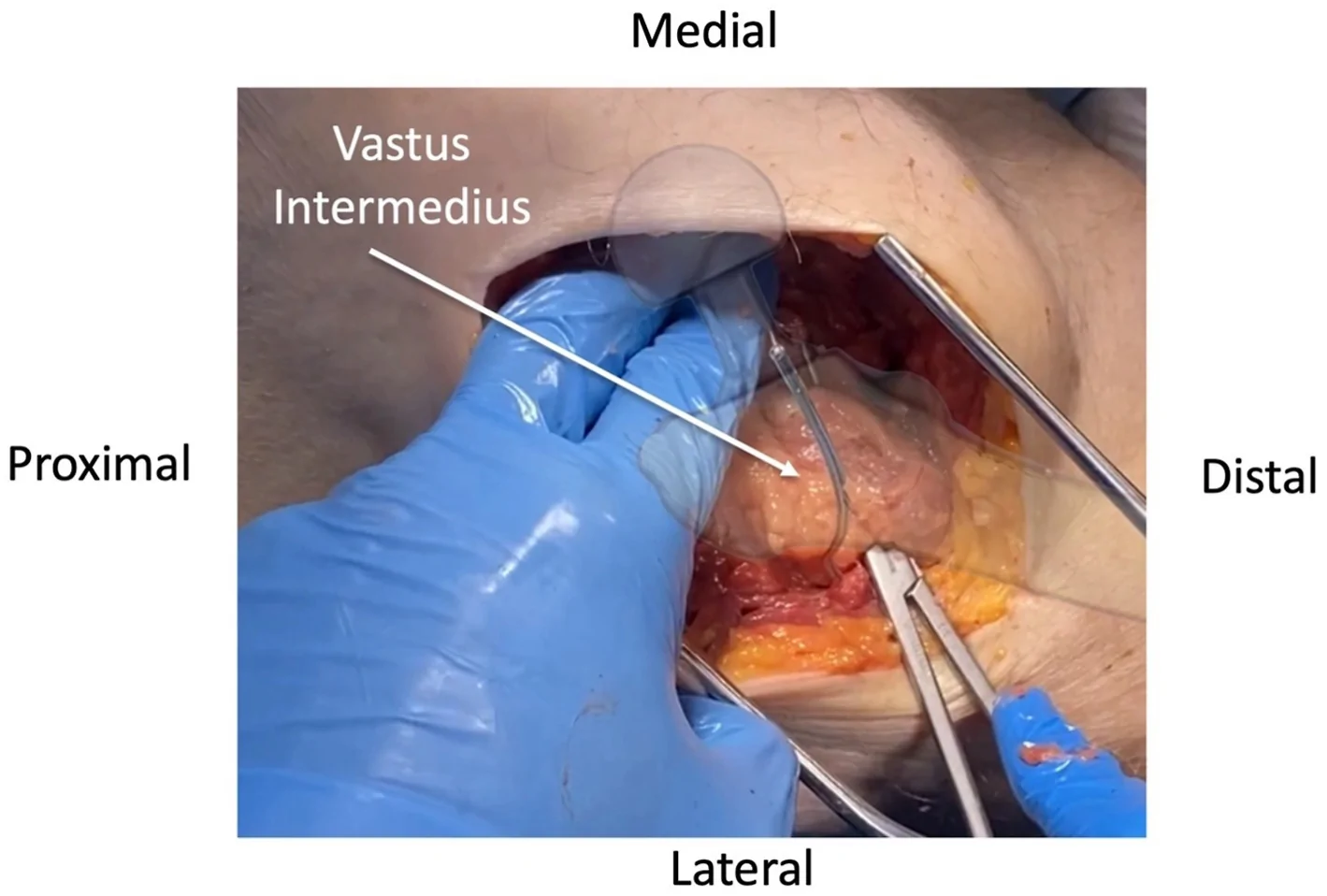
Intraoperative photograph demonstrating retrieval of the looped wire end from the medial side using a tonsil clamp passed under the vastus intermedius.

A ball-tipped wire or cable is preferred, as the smooth ball end is less likely to snag tissue. Insert the ball-tipped end into the hole in the tip of the cable passer, wedging it securely. Next, return the leg to a neutral position to relax the muscles. At this stage, the wire has been carried around the back of the femur and out anterolateral, deep into the vastus lateralis. Its ball-tipped end should be at or near the anterolateral side of the femur deep into the vastus intermedius muscle; secure this with a clamp to avoid losing it. To retrieve the medial looped tail of the wire, pass the tonsil clamp from the lateral side of the femur under the vastus intermedius to medial, grasping the looped end of the wire and pulling the wire to the lateral side underneath the vastus intermedius (Fig. 6). Now with the wire encircling the femur, ensure it lies in the desired position on bone and just proximal to the lesser trochanter (LT) as desired for a proximal femoral cerclage. There should be minimal soft tissue interposed between the wire and bone.

With both ends of the cerclage wire now accessible on the lateral side, tension and secure the cerclage in the standard fashion for the chosen implant until it is snug around the anteromedial calcar, avoiding over-tensioning to the point of implant or bone breakage. Once the desired tension is achieved, cut the excess wire using wire cutters or the cable using a cable cutter. Typically, the wire is left with 3-4 twists, and then using a large needle driver, the end is turned clockwise down against the femur in a final tightening twist. The goal is to bury the twisted wire knot deep under the vastus intermedius muscle away from the tensor fascia or subcutaneous tissue to minimize the risk of soft-tissue irritation or impingement. Examine the surrounding area to ensure no muscle or neurovascular structure is entrapped or impinged by the wire. Hemostasis should be confirmed, as passing instruments can sometimes disrupt small vessels, even though we have found using the blunt cable passer mitigates this risk.

### Additional cerclage technique

**Figure 7:**
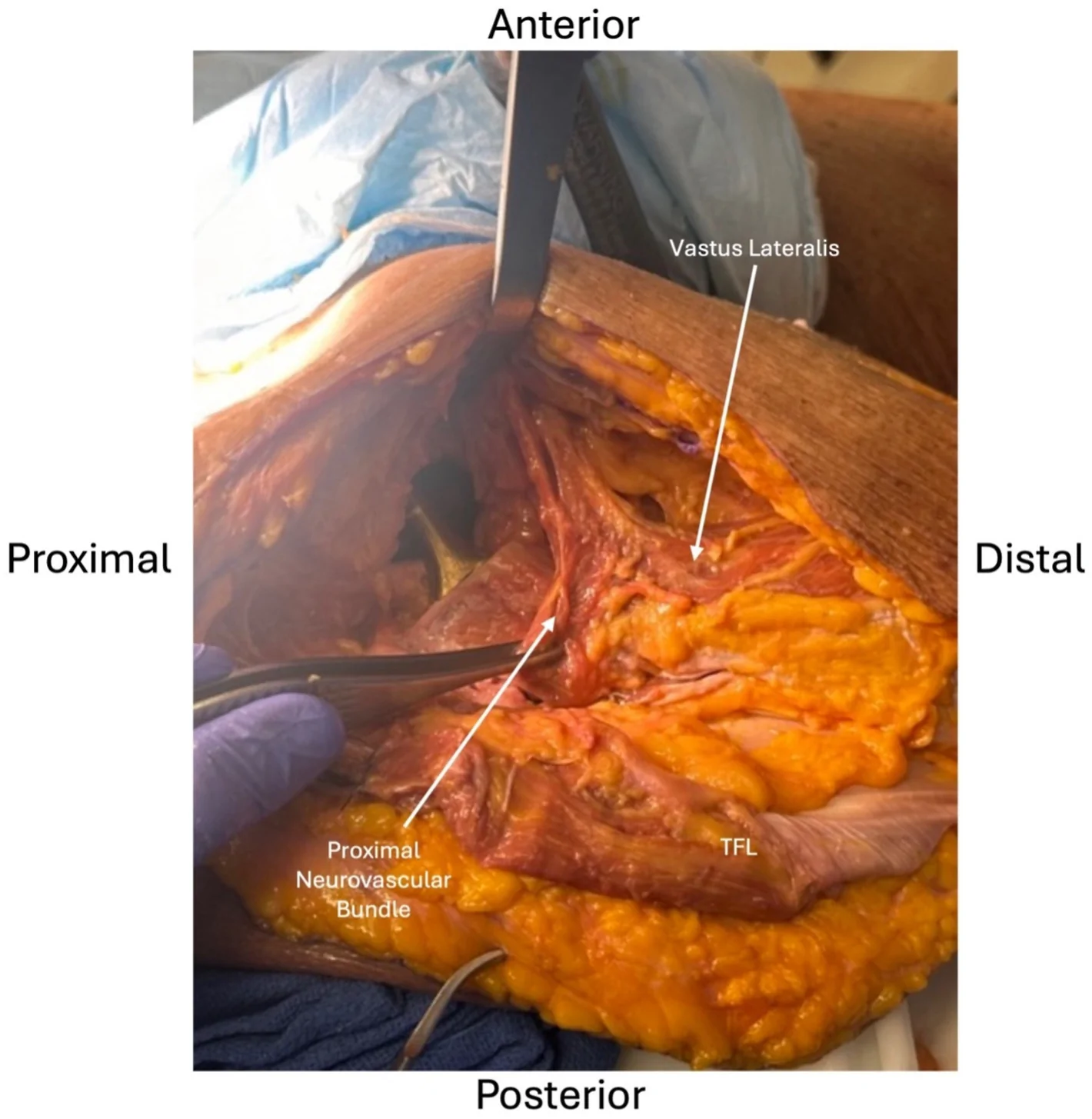
Intraoperative photograph demonstrating the proximal neurovascular bundle crossing over the anterior femur within 2 cm of the lesser trochanter. TFL, tensor fascia lata.

If additional wires are required, the steps as described earlier can be used with a few additional considerations. When passing a cerclage wire below the LT, one must be cautious of the proximal neurovascular bundle (PNVB) containing the transverse branch of the LFCA and proximal main branch of the motor nerve to the vastus lateralis (Fig. 7). Depending on the height of the calcar and relative position of the PNVB, a wire can be placed below the LT carefully while avoiding the PNVB. Previous cadaveric dissection studies have demonstrated that the PNVB crosses 1.6 ± 0.5 cm from the LT [4]. To pass another wire further distal to the LT, the safest window exists distal to the PNVB.

**Figure 8:**
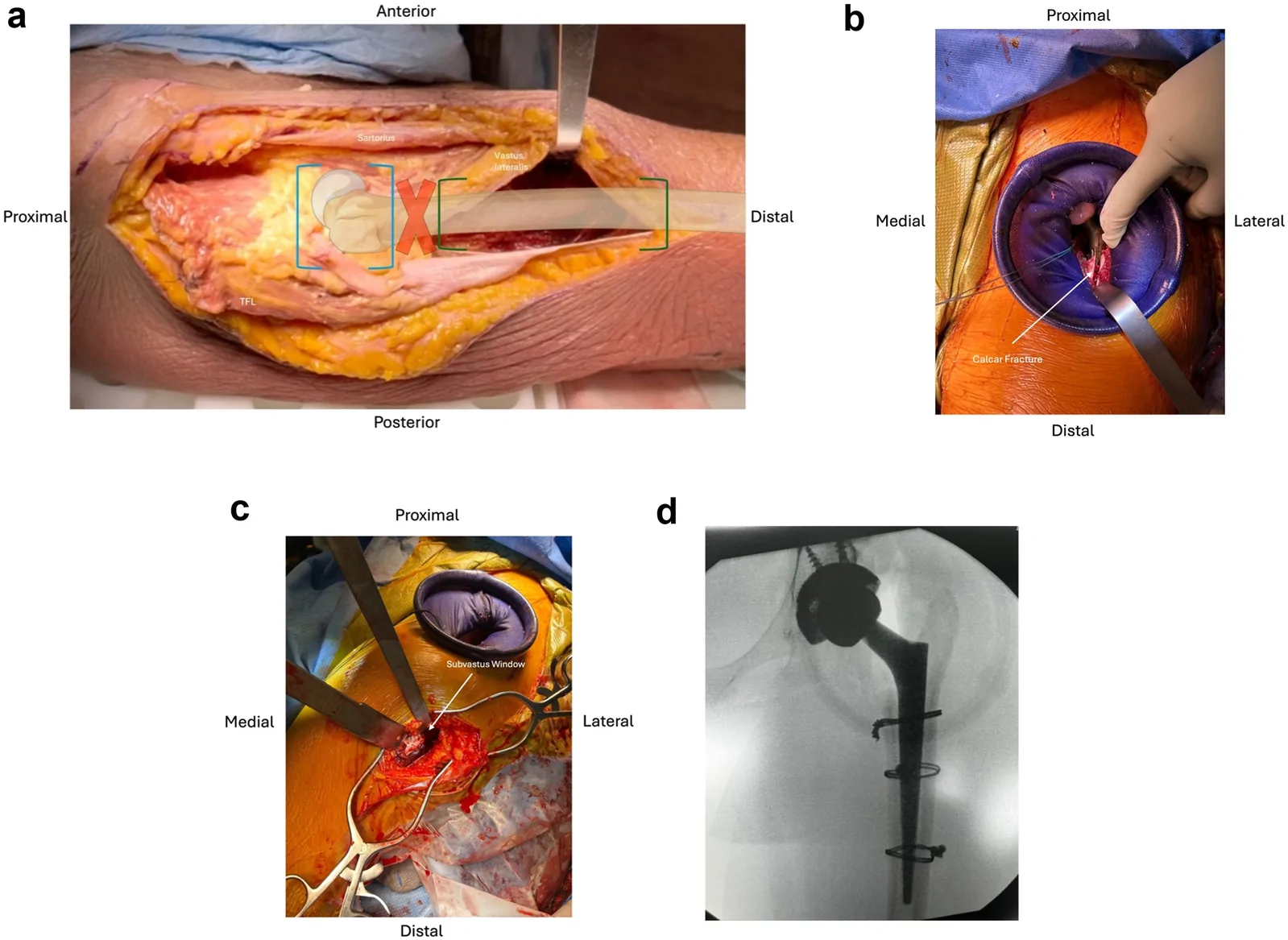
(a) Cadaveric specimen demonstrating an extensile DA incision to access the subvastus window for distal wire passage. Blue brackets indicate the DA wire passing the safe zone, green brackets indicate the lateral/subvastus exposure safe zone, and the red X marks where the PNVB crosses the LT. (b) Intraoperative photograph demonstrating an intraoperative calcar fracture. (c) Intraoperative photograph demonstrating a separate lateral incision technique to access the subvastus window for distal wire passage. (d) Anteroposterior radiograph demonstrating final placement of distal cerclage wires.

**Figure 9:**
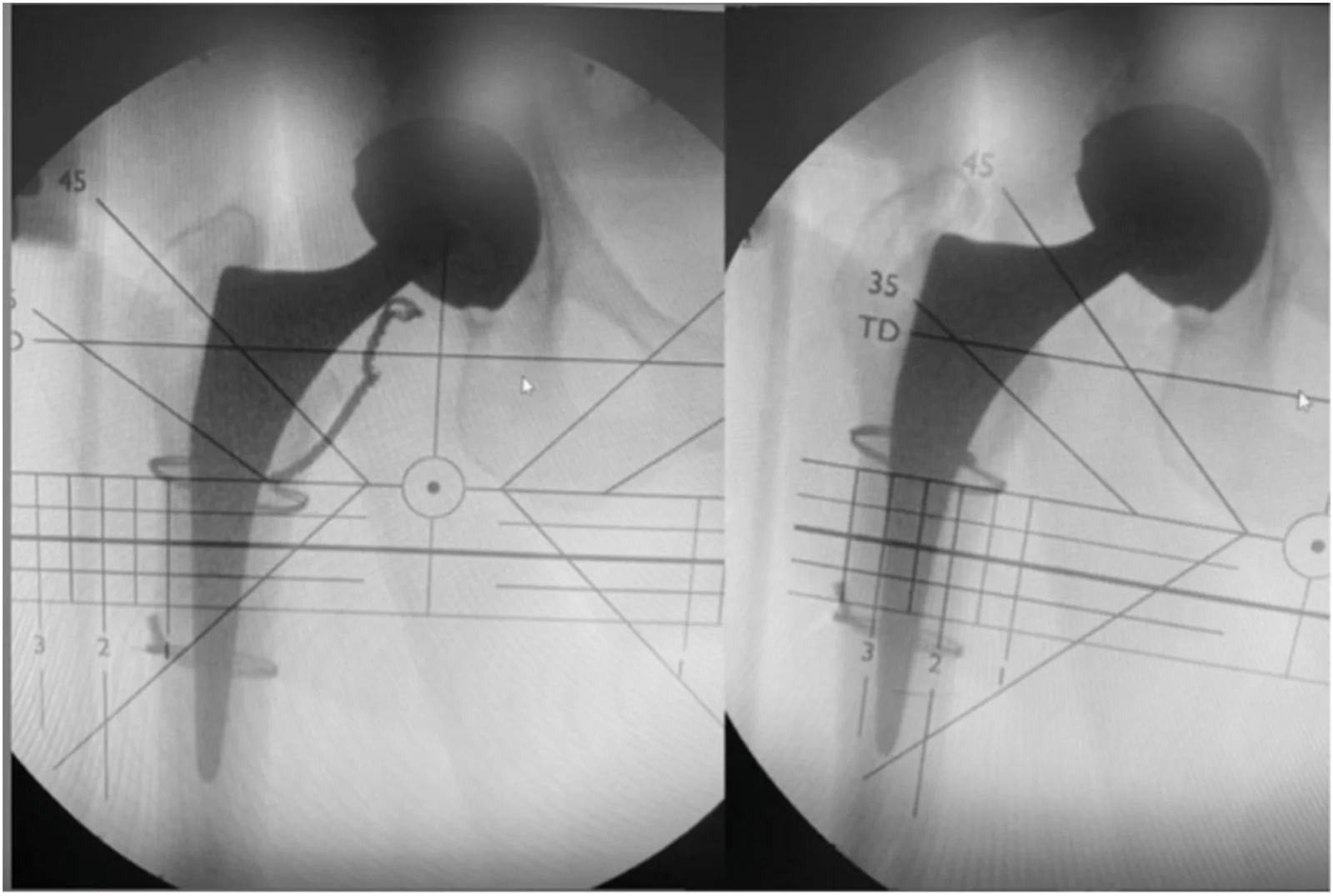
Intraoperative fluoroscopic image demonstrating a calcar fracture with 2-cm extension below the LT, treated with cerclage wires placed above and below the LT.

**Figure 10:**
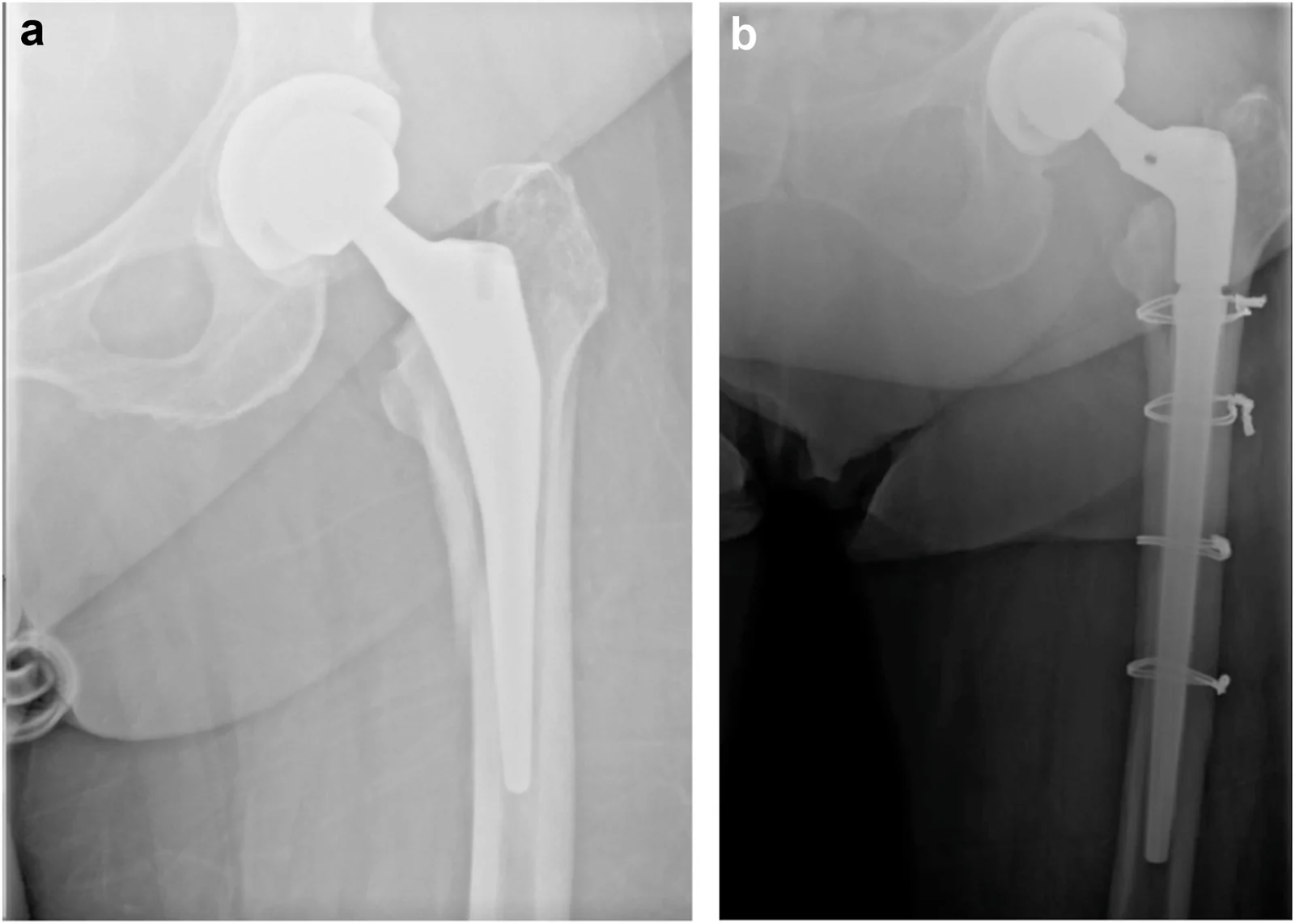
Anteroposterior pelvis radiographs demonstrating (a) a Vancouver B periprosthetic fracture and (b) treatment with a splined tapered stem secured with multiple cerclage wires placed using the described technique.

If additional distal wires are needed, exposure of the femur requires either an extensile DA approach with an extension into a subvastus approach or a separate subvastus ancillary incision can also be utilized (Fig. 8). After extending the incision or making a separate incision on the lateral aspect of the femur, the iliotibial band is incised in line with the femur. The vastus lateralis is elevated from the femur, and then the previously described technique can be used to pass wires around the femur with manipulation of the leg. When using a bikini incision to access the DA interval, the authors find the use of a separate incision to be safer and less invasive than extending it into an extensile DA approach (Figs. 9 and 10).

## Discussion

This article presents a simple and reliable technique for passing cerclage wires during the direct-anterior-approach THA. By utilizing a nonhooked cable passer and a submuscular trajectory, the surgeon can safely encircle the proximal femur without the risk of disrupting vasculature. The method is reproducible and does not require special access incisions. The same technique can be used with either wires or cables, according to surgeon preference and the demands of the case. The technique described is straightforward to learn, especially for surgeons already familiar with the anterior approach anatomy.

Safety is a key advantage of this method. The blunt passer avoids the pitfalls of the classic hooked wire passer, making it significantly less likely to injure a perforating vessel in the anterior thigh and avoid snagging of the proximal and distal neurovascular bundles to the vastus lateralis. By carefully maintaining contact with the femur and using strategic leg positioning, the wire can be passed along a safe plane every time. The choice of cerclage material—wire, metal, cable, or polymeric cable—is left to the surgeon, as the passage technique described here is identical for each.

## Summary

Cerclage wire passage in DA THA is not only doable but also safe and efficient with the described technique. This achieves cerclage fixation in the DA approach with equivalent effectiveness to traditional methods, while offering improved safety. Adopting a nonhooked cable passer and following the stepwise procedure outlined earlier allow the surgeon to quickly secure the femur when needed, whether prophylactically preventing or treating an intraoperative calcar fracture or securing a controlled anterior extended osteotomy or window osteotomy. This technique emphasizes simplicity and reproducibility and avoids undue trauma to musculature or neurovascular bundles. Surgeons can confidently add this wire-passing method to their anterior approach skill set, knowing that it provides a secure option for cerclage fixation with minimal risk.

## CRediT authorship contribution statement

**Nikhil Vallabhaneni:** Writing – review & editing, Writing – original draft. **Utkarsh Anil:** Writing – review & editing, Conceptualization. **Allan Metz:** Writing – review & editing, Writing – original draft. **Jeremy M. Gililland:** Writing – review & editing, Supervision, Conceptualization. **Lucas A. Anderson:** Writing – review & editing, Supervision, Conceptualization.

## Conflicts of interest

Dr. Gililland receives royalties from Zimmer Biomet and Stryker. He is a consultant for Stryker, Enovis, and Zimmer Biomet. He possesses stock in CoNextions, MiCare Path, Sylke, Solenic, and OR Innovations. He receives research support from Stryker, Zimmer Biomet, and Medacta. He sits on the editorial board for the *Journal of Arthroplasty* and is part of the American Association of Hip and Knee Surgeons program committee. Dr. Anderson has been a paid speaker for Medacta in addition to being a paid consultant. He owns stock in OrthoGrid and received research support from Stryker and Zimmer Biomet. He is a section editor of the Société Internationale de Chirurgie Orthopédique et de Traumatologie for hip and knee arthroplasty. All other authors declare no potential conflicts of interest.

For full disclosure statements refer to https://doi.org/10.1016/j.artd.2026.102146.

## Appendix A Supplementary data

Conflict of Interest Statement for Anderson

Conflict of Interest Statement for Anil

Conflict of Interest Statement for Gililland

Conflict of Interest Statement for Metz

Conflict of Interest Statement for Vallabhaneni
